# Is there a relationship between the morphology of the forewing axillary sclerites and the way the wing folds in aphids (Aphidomorpha, Sternorrhyncha, Hemiptera)?

**DOI:** 10.1007/s00435-017-0390-7

**Published:** 2017-11-30

**Authors:** Barbara Franielczyk-Pietyra, Tytus Bernas, Hanna Sas-Nowosielska, Piotr Wegierek

**Affiliations:** 1 Department of Zoology, Faculty of Biology and Environmental Protection, University of Silesia, Katowice, Poland; 2 Laboratory of Imaging Tissue Structure and Function, Nencki Institute of Experimental Biology, Warsaw, Poland

**Keywords:** Aphids, Axillary sclerites, Wing base, Wings folding

## Abstract

The present study describes the relationship between the morphology of the forewing axillary sclerites and the way the wings fold among 24 aphid genera as compared to a representative of coccids. Architecture of the forewing base was imaged with scanning electron and optical (fluorescence) microscopy. Significant differences in morphology of axillary sclerites between aphid species were observed, despite their belonging to one infraorder. Detailed description of 41 features of axillary sclerites was made. There was no difference between axillaries of viviparous (Aphididae) and oviparous (*Adelges* sp., *Phylloxera* sp.) species. No clear relationship between morphology of the axillary sclerites and the wing folding could be confirmed. Instead, the thorax structure determines the way the wing folds in aphids. Phylogenetic analysis based on our results cannot be conducted at this stage of study. To show how three-dimensional the structures are and how difficult to describe, a short animation of *Aphis fabae* (Aphididae) wing base was added. This is a preliminary study about morphology of axillary sclerites among aphids.

**Electronic supplementary material:**

The online version of this article (10.1007/s00435-017-0390-7) contains supplementary material, which is available to authorized users.

## Introduction

**Fig. 1:**
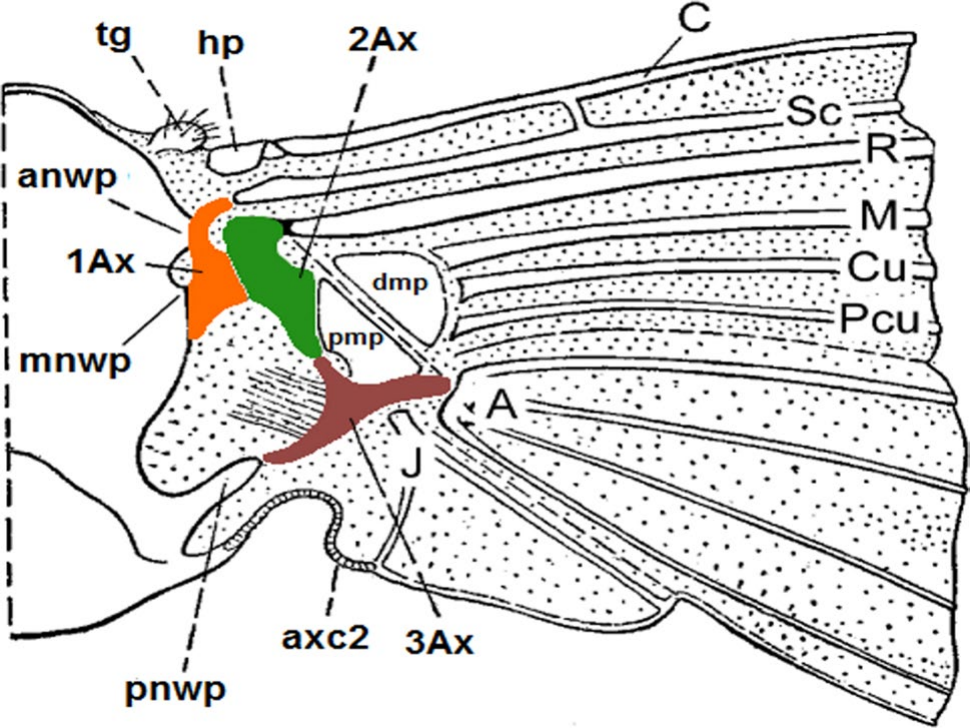
Model of the insect wing articulation. After Snodgrass (1935), modified; abbreviations in the text

The wing base and membrane form a complex structure which is responsible for the ability of insects to fly. The structure comprises several cooperating elements, such as axillary sclerites and muscles (Fig. 1).

The axillary sclerites, which usually are composed of three pieces (Snodgrass 1935), have the same ground-plan. The first sclerite (1Ax) is a longitudinal element that is composed of the head, neck and the basal part—the body. There is an *α* angle between the proximal and distal leg of 1Ax (Hörnschemeyer 1998). This sclerite articulates the anterior notal wing process (anwp). The second sclerite (2Ax) has a triangular shape and has a wide projection on the ventral side that turns underneath and reaches 1Ax. The last sclerite, 3Ax, is longitudinal and is situated between the proximal notal wing process of the notum (pnwp) and the wing membrane (Hörnschemeyer 2002). All of these sclerites are connected together by a system of very thin, barely visible membranes, which permit wing folding while at rest.

**Fig. 2:**
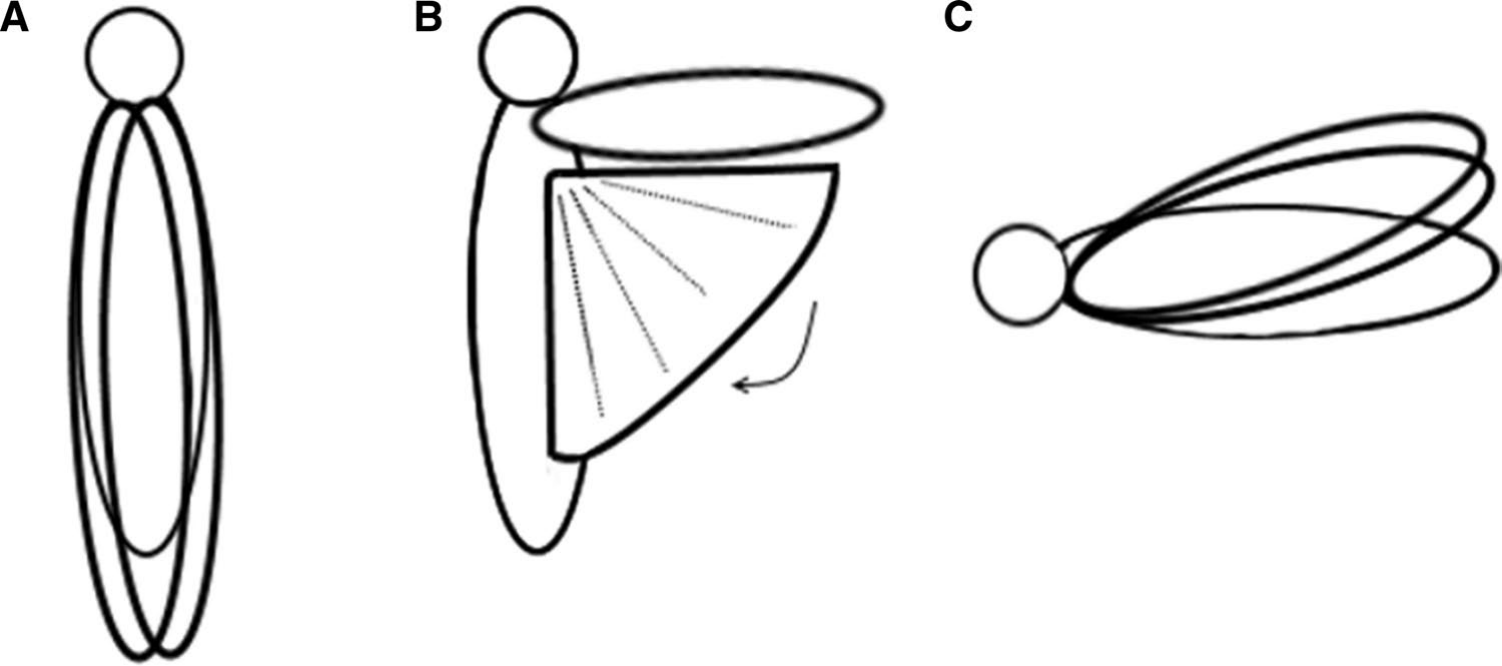
Schematic drawings of the ways that insect wings fold: **A** flat, **B** outlining, **C** roof-like

According to Brodsky (1996), there are three ways that insects fold their wings. Insects can fold their wings flat (over the abdomen and overlapping each other), outlining (the hindwings are bent longitudinally and folded fan-like under the forewings) or roof-like (the wings do not overlap and only posterior margins have contact) (Fig. 2).

The aphids described in this study belong to Aphidomorpha (Becker-Migdisova and Aizenberg 1962), one of the infraorders of Sternorrhyncha. The remaining infraorders are: Coccomorpha (scale insects) (Heslop-Harrison 1952), Psyllomorpha (jumping plant-lice = psyllids) (Becker-Migdisova and Aizenberg 1962) and Aleyrodomorpha (whiteflies = aleyrodids) (Chou 1963). Two pairs of wings are characteristic for some aphids, most psyllids and whiteflies adults, while only a single pair of wings is well-developed in male scale insects (Gullan and Martin 2009).

Aphids were chosen as the model group for this study for several reasons. First of all, aphids are the earliest Sternorrhyncha group that has been found in fossil material (Permian, Triassic) (Hong et al. 2009; Shcherbakov 2010; Heie and Wegierek 2011; Szwedo et al. 2015). Secondly, aphids have the highest taxonomic diversity (5100 species) (Favret 2016). Moreover, many distinguishing features such as various degrees of the wing usage in translocation or different model of wings arrangement at rest—roof-like (as in psyllids) or flat over abdomen (as in coccids) (Miyazaki 1987) can be observed in this group of insects. In addition, aphids have significant modifications of their wing venation that ranges from full (as in psyllids) to strongly reduced or restricted to single veins (as in coccids and aleyrodids). The different relationships between the forewing and hindwing in Sternorrhyncha is also worth mentioning. Among aphids, there is an entire spectrum of variation that ranges from both pairs being proportional to a strongly reduced hindwing (as in coccids).

Here we present (1) relationship between the axillary structure and the types of wing folding; (2) comparison of the morphology of the axillary sclerites between species from the Aphidomorpha infraorder.

## Materials and methods

### Taxa examined and terminology

The taxa of 24 genera from Aphidomorpha (alate morphs) that were examined in this study are listed in Online Resource 1. Most specimens were collected in Poland, except for *Hormaphis* sp., *Greenidea* sp.—South Korea and *Neuqenaphis* sp.—South America (Chile). No specific permissions were required for these locations because specimens were collected from public places, not from private properties or protected areas. The field studies did not involve endangered or protected species. For comparison, *Orthezia urticae*, a representative of Coccomorpha, was selected as the sister group of Aphidomorpha according to genetic (von Dohlen and Moran 1995; Xie et al. 2008) and morphological studies (Hennig 1981; Carver et al. 1991). Moreover, the wing of *O. urticae* is considered to be the most primitive among the known scale insects (Shcherbakov 2007). *Adelges* sp., which is a taxon belonging to extant oviparous aphids that are considered to be more primitive, as compared to numerous viviparous aphids, was also selected (Nováková et al. 2013). Based on their biology (Heie 1987) and on molecular (Nováková et al. 2013) and anatomical (Szklarzewicz et al. 2000) studies (oviparity), this taxon is rich in plesiomorphic characters.

The terminology of the wing base structures follows Hörnschemeyer (2002) and Yoshizawa and Saigusa (2001). The abbreviations used in the text and in the figures are:

anwp—anterior notal wing process; 1Ax, 2Ax, 3Ax—axillary sclerites 1, 2, 3; axc2—axillary cord; dmp—distal median plate; hp—humeral plate; mnwp—median notal wing process; pmp—proximal median plate; pnwp—posterior notal wing process; psc2—prescutum; psc2 + sc2—prescutum fused with mesoscutum; sc2—mesoscutum; scl2—mesoscutellum; tg—tegula.

Some of presented axillary characters were based on Zhao et al. (2014).

### Dissection and examination

Specimens were prepared according to the method described by Kanturski and Wieczorek (2012) using 10% KOH, chloro-phenol and chloral hydrate after which the axillary sclerites were removed from the body and observed in glycerin under Nikon SMZ1500 stereomicroscope. All drawings were done from the right forewing and the orientation of the axillaries that are described is in relation to the main axis of the body. This perspective has been chosen to present the interspecies differences in the most comprehensive manner.

For scanning electron microscope (SEM) analysis, specimens of *Aphis fabae* were dehydrated in a graded series of increasing concentrations of ethanol (75–100%) and transferred to 100% HMDS. Next, the specimens were mounted on holders, sputter-coated with gold and examined using a Hitachi UHR FE-SEM SU 8010 scanning electron microscope (Tokyo, Japan) in the Scanning Electron Microscopy Laboratory at the Faculty of Biology and Environmental Protection, University of Silesia.

A similar protocol was used for the SEM analysis of *Thelaxes* sp., but sputter-coating with gold has been omitted because the insects had been examined using a COXEM EM-30 scanning electron microscope (Korea) at the Department of Zoology, University of Silesia.

For imaging using fluorescence microscopy, the specimens were prepared using a standard protocol (Kanturski and Wieczorek 2012), stained with acridine orange and embedded in 1% agarose solution. Zeiss MP7 multiphoton microscope equipped with 20x/1.0 water immersion objective, TiSa pulsed laser (Chameleon, Coherent) and GaAsP detectors was used for the imaging (Laboratory of Imaging Tissue Structure and Function, Nencki Institute of Experimental Biology, Warsaw). Acridine orange fluorescence was excited using a 750 nm laser wavelength and was detected in the 500–550 nm and 570–610 nm ranges. Image analysis and 3D reconstructions were performed using Imaris (Bitplane) software.

**Fig. 3:**
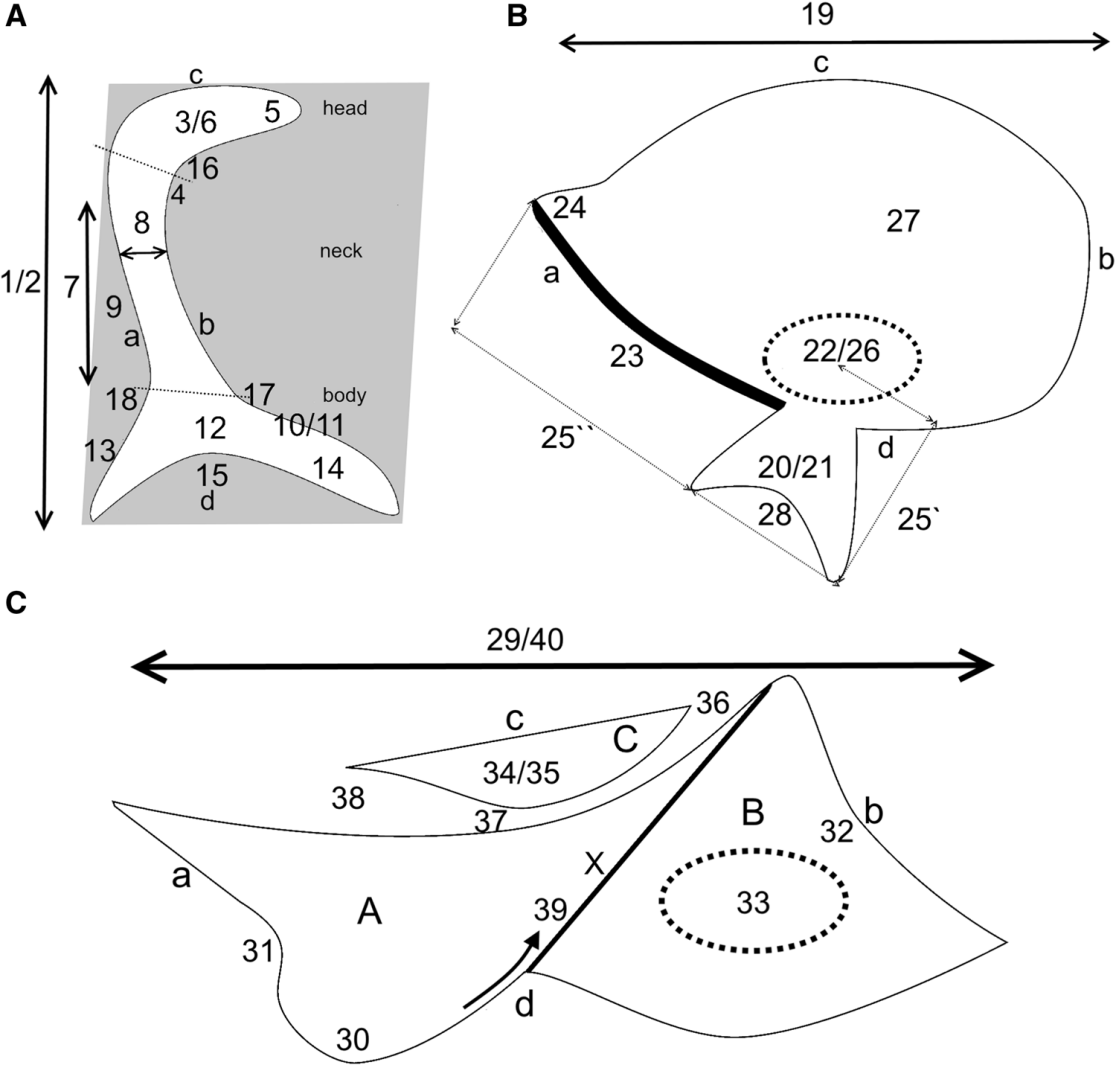
Overall model of the axillary sclerites for aphids: **A** 1Ax, **B** 2Ax, **C** 3Ax

All of described features were indicated on schemas of the axillary sclerites (Fig. 3). The boundary between the head and neck of 1Ax was designated by a plane parallel to the body of the axillary, or if it could be identified, by the indentation between the head and neck membrane (maintaining a parallel plane). The boundary between the neck and the body was determined by a tangent line to the body plates that passed perpendicularly through the neck. All of the axillaries had defined proximal, distal, anterior and posterior sides (in sequence A, B, C, D on Fig. 3A–C).

The results for three axillary sclerites that build the forewing base in aphids are presented here. The ground-plan of the entire wing base was discussed in a previous work (Franielczyk and Wegierek 2016).

## Results

**Fig. 4:**
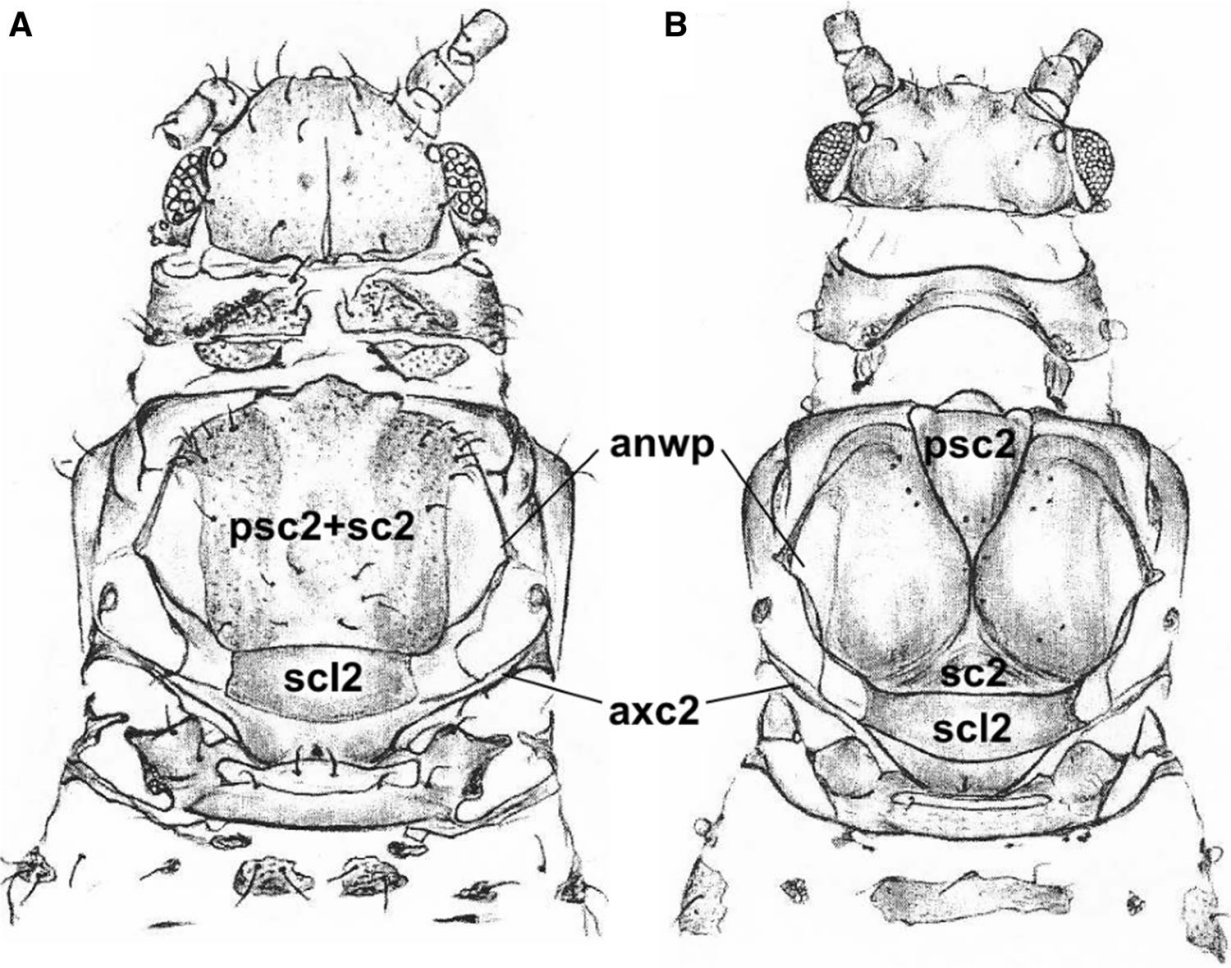
Schematic drawings of the thorax: **A** *Glyphina betulae* Linnaeus, 1758 (Thelaxidae); **B** *Aphis fabae* Scopoli, 1763 (Aphididae); after Wegierek (2002), modified

**Fig. 5:**
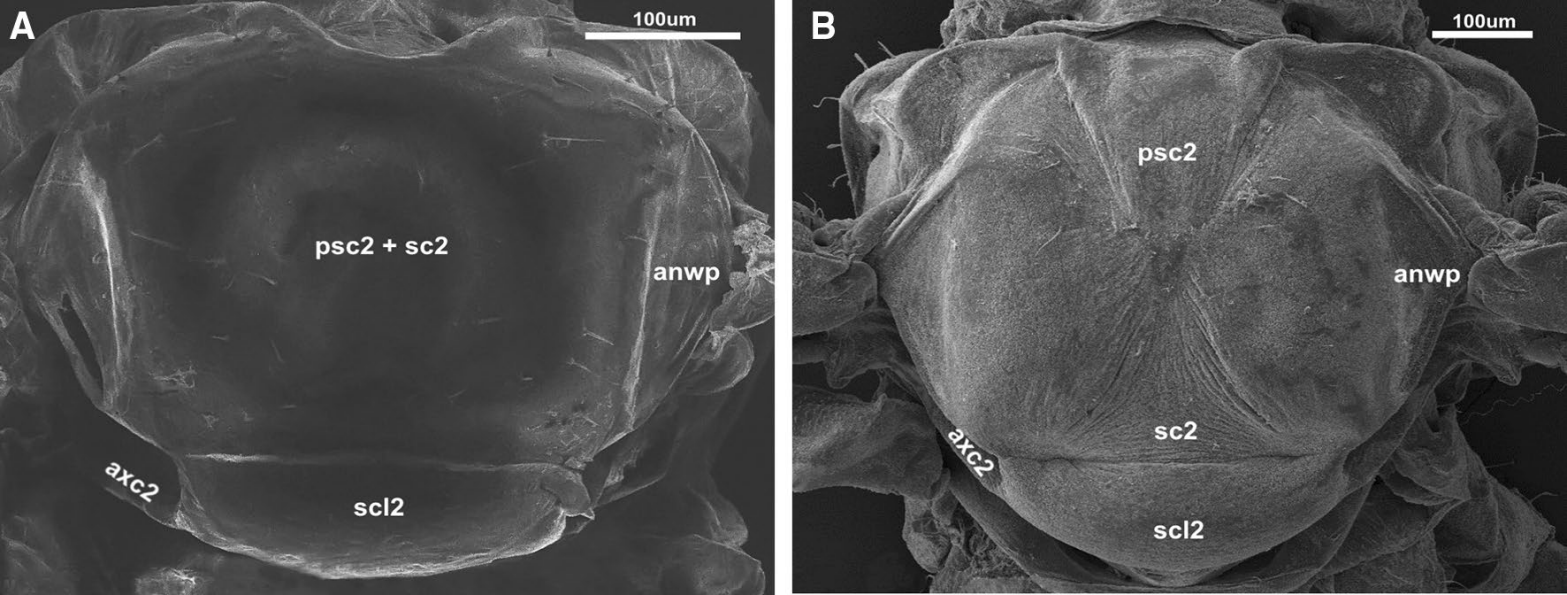
Scanning electron microscopy showing the thorax: **A** *Thelaxes* sp. Westwood 1840 (Thelaxidae); **B** *Aphis fabae* Scopoli, 1763 (Aphididae)

Most aphids differ slightly with respect to the external morphology of the thorax. The prescutum (psc2) may be much smaller than the mesoscutum (sc2) when it is composed of two strongly sclerotized plates that are usually convex. Such a condition is especially visible in aphids that fold their wings roof-like when at rest (Fig. 4B). However, when the wings are folded flat at rest, the bulges of the thorax are more (Phylloxeridae) or less clear (Phloeomyzidae, Thelaxidae) (Brodsky 1996). In that type of thorax, the prescutum and scutum are fused together (Fig. 4A). Those elements are limited by the anterior notal wing processes (anwp) on both sides. The scutellum is situated under the prescutum (scl2). In both types of wing folding, this structure is rectangular in shape (Fig. 5A, B) and is connected to the axillary cord (axc2).

### Characters description of the forewing axillary sclerites to show morphological variations between examined genera

#### First axillary sclerite (1Ax) (Fig. 3A)

1. Shape of axillary is: (a) trapezium; (b) trapezoid; (c) parallelogram.
2. Sclerotization of the entire axillary: (a) very strong; (b) weak.
3. Head is: (a) elongated (≥ 30 µm) and parallel to the body; (b) short (< 30 µm) and parallel to the body; (c) shortened.
4. A membrane supports both the head and neck: (a) strongly; (b) slightly; (c) lack of membrane.
5. The ending of the head of 1Ax is: (a) sharp; (b) rounded; (c) extended.
6. The head is: (a) curved down; (b) parallel to the body; (c) curved up.
7. The length of the neck of 1Ax is: (a) shorter than the head of 1Ax; (b) as long as the head of 1Ax; (c) longer than the head of 1Ax.
8. The width of the neck of 1Ax is: (a) the same width along the entire length; (b) narrower at the beginning behind the head; (c) wider at the beginning behind the head.
9. The neck in 1Ax: (a) is slightly bent outward; (b) forms a single plane with the body; (c) is strongly bent outward.
10. The outgrowth of the body of 1Ax is: (a) present closer to neck; (b) absent; (c) present further from the neck.
11. The location of the body’s outgrowth (if present) is: (a) on the dorsal side of the body; (b) on the ventral side of the body.
12. The body consists of: (a) uniform elements; (b) two thin legs connected by membrane.
13. A proximal outgrowth of the body is: (a) absent; (b) present.
14. The length of the body legs: (a) is equal; (b) the distal leg is longer than proximal.
15. The angle between the legs of the body are: (a) more than 50°; (b) less than 50°.
16. The angle between the head and neck is: (a) 90°; (b) more than 90°; (c) less than 90°.
17. The internal angle between the neck and distal leg is: (a) more than 50°; (b) less than 50°.
18. The external angle between the neck and proximal part of the body is: (a) less than 100°; (b) more or equal to 100°.

#### Second axillary sclerite (2Ax) (Fig. 3B)

19. The shape of 2Ax is: (a) triangular; (b) oval; (c) trapezoid.
20. A ventral outgrowth is: (a) present; (b) absent.
21. The ventral outgrowth (when present) is sclerotized: (a) strongly; (b) weakly.
22. The connection between the ventral outgrowth and the middle of the axillary is: (a) strongly sclerotized; (b) weakly sclerotized.
23. The proximal edge of the axillary is: (a) thin; (b) thick.
24. The proximal edge of 2Ax ends: (a) by smoothly passing through the rest of the membrane; (b) by the membrane being curved upward on the outside.
25. The relation between the length of the proximal edge (25″) of 2Ax and the length of the ventral outgrowth (25′): (a) the ventral outgrowth is shorter than the proximal edge (25′ < 25″); (b) is the same length (25′ = 25″).
26. The site of the rounded element on the axillary is: (a) in the middle; (b) closer to the proximal edge; (c) closer to the distal edge.
27. The membranous part of the axillary creates: (a) a bowl-like indentation; (b) a flat surface.
28. The ventral outgrowth: (a) is not surrounded by the membrane; (b) is surrounded by the membrane; (c) surrounds the membrane.

#### Third axillary sclerite (3Ax) (Fig. 3C)

29. The shape of 3Ax is: (a) trapezoid; (b) triangular.
30. The posterior membrane of 3Ax is: (a) present; (b) absent.
31. The shape of the margin of the posterior membrane is: (a) plain; (b) indented.
32. The distal part of the axillary ends as: (a) thick arms; (b) thin arms.
33. The shape of the distal aperture in the membrane is: (a) narrow; (b) wide.
34. The presence of an anterior outgrowth is: (a) present; (b) not present.
35. The bowl-like anterior outgrowth is: (a) short; (b) elongated.
36. The site of the anterior outgrowth is: (a) not very close to the edge of the membrane near the distal aperture; (b) very close to the edge of the membrane near the distal aperture.
37. The connection between the anterior outgrowth and the anterior part of the axillary is: (a) visible; (b) not visible.
38. The connection between the proximal edge of the anterior outgrowth and the proximal part of 3Ax is: (a) no connection; (b) by a thin membrane; (c) by a wide membrane.
39. The edge of the membrane in the middle of the axillary: (a) extends upwards to the ventral side; (b) extends flat to the ventral side.
40. The proximal and distal parts of the axillary: (a) the proximal part is longer than the distal part; (b) are the same length; (c) the proximal part is shorter the distal part.
41. The way that the wings fold is: (a) roof-like; (b) flat.

### Axillary sclerites

#### First axillary sclerite 1Ax (Fig. 3A—numbers in curly brackets; Fig. 6)

**Fig. 6:**
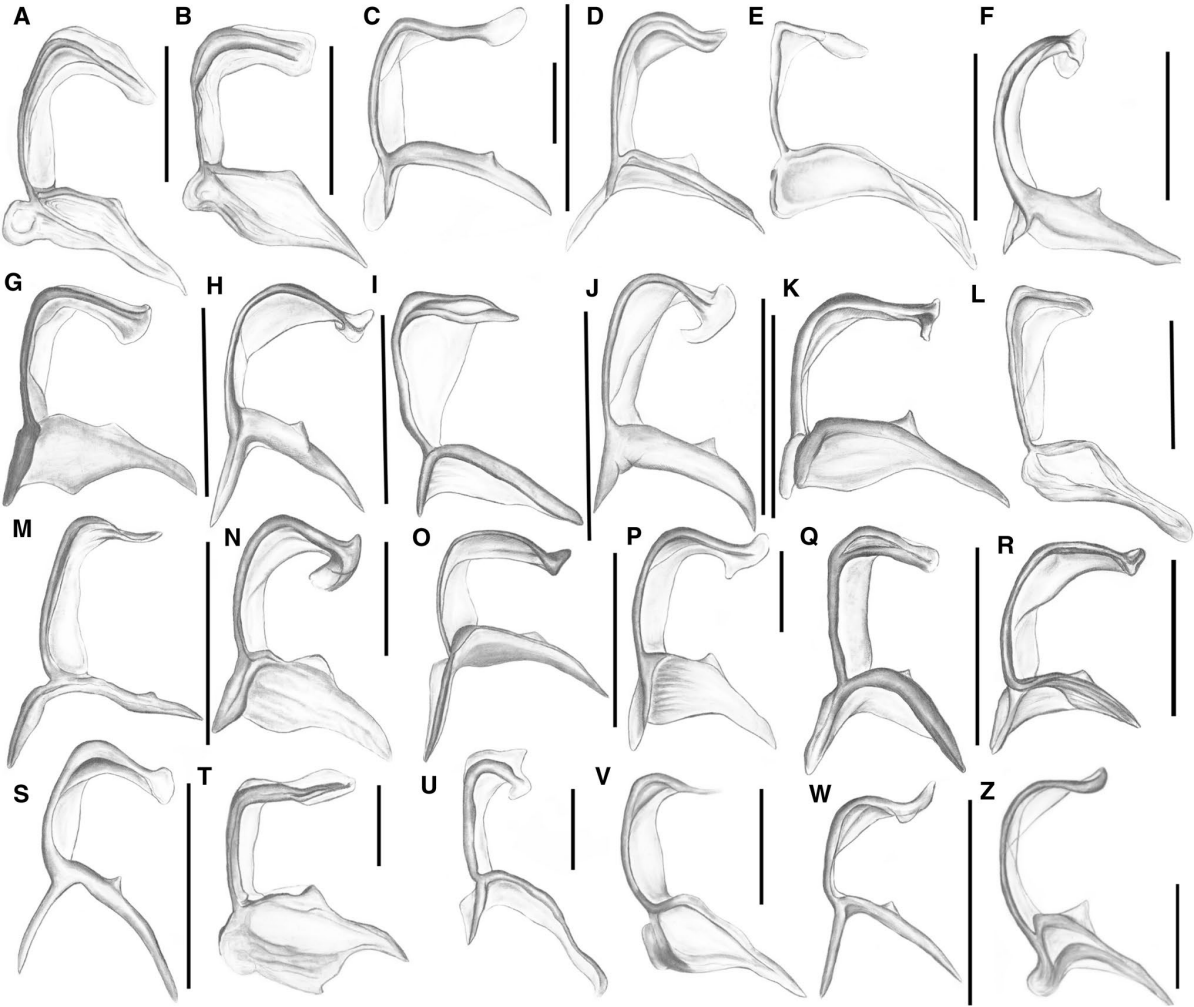
Schematic drawings of 1Ax: **A** *Adelges* sp.; **B** *Anoecia* sp.; **C** *Aphis* sp.; **D** *Chaitophorus* sp.; **E** *Cinara* sp.; **F** *Drepanosiphum* sp.; **G** *Eriosoma* sp.; **H** *Eucallipterus* sp.; **I** *Glyphina* sp.; **J** *Greenidea* sp.; **K** *Hormaphis* sp.; **L** *Lachnus* sp.; **M** *Macrosiphum* sp.; **N** *Mindarus* sp.; **O** *Neuqenaphis* sp.; **P** *Pemphigus* sp.; **Q** *Phloeomyzus* sp.; **R** *Phylloxera* sp.; **S** *Phyllaphis* sp.; **T** *Prociphilus* sp.; **U** *Thelaxes* sp.; **V** *Trama* sp.; **W** *Tuberculatus* sp.; **Z** *Tuberolachnus* sp.; scale bar 0.025 mm, except for (*R*) = 0.0125 mm

In outline 1Ax looks like a trapezium (Fig. 6A, F, I, J, K, P, Z), trapezoid (Fig. 6B, D–E, G, H, L–O, Q–W) or parallelogram (Fig. 6C) {1}. The sclerotization of the entire sclerite can vary from strong (Fig. 6A–C, F–I, K, M–R, T, W–Z), to weak (Fig. 6D, E, J, L, S, U, V) {2}. The three parts of the first sclerite that can be distinguished and are easiest to recognize are the head, neck and body. The head can be elongated (≥ 30 µm) and parallel to the body (Fig. 6A–D, G–I, K, N–P, R–T, W–Z), short (< 30 µm) and parallel to the body (Fig. 6E, J, L-M, Q, U-V) or shortened (Fig. 6F) {3}. The position of the head may be parallel to the body (Fig. 6B–D, G, H, K, M, O, P, T, Z) or may be curved—upwards (Fig. 6I, S, W) or downwards (Fig. 6A, E, J, L, N, Q, R, U, V) {6}. Endings of the head may be rounded (6C, 6L, 6Q, 6S), sharp (6A, 6E, 6I, 6M, 6T, 6V-Z) or extended (Fig. 6B, D, F–H, J, K, N–P, R, U) {5}. There is a weak (Fig. 6E, K, W–Z) or strong membrane (Fig. 6A–D, F–J, L–V) between the head and neck {4}. The second element, the neck, may vary in length. In most cases, it is as long as the head (Fig. 6O), but it may also be longer (Fig. 6M, Q) or shorter (Fig. 6A–L without F, 6N, 6P, 6R-Z) than the head {7}. The width of the neck is also a differentiating feature—it may have the same width along its entire length (Fig. 6A–E, K, N, P–Z) or it may be narrower (6M, 6O) or wider (6G-J, 6L) at the beginning {8}. The neck may also be bent slightly (Fig. 6A, C, E, G–J, N, P) or strongly outward (6F, 6O, 6S, 6V-Z) or it may form a single plane with the body (Fig B, 6D, 6K-M, 6Q-R, 6T-U) {9}. The last part of 1Ax, the body, consists of a uniform element (Fig. 6A–B, E–G, K, L, N, P, T, V, Z) or two thin legs (Fig. 6C, D, H–J, M, O, Q–S, U, W) that are connected by a membrane {12}. The lengths of the body legs may be equal or the distal one may be longer (Fig. 6C, D, H–J, M, O, Q–S, U, W) {14}. Two outgrowths can be distinguished on the body—one on the proximal part of the body (Fig. 6A, B, K, V, Z) {13} and the second on the distal part (Fig. 6A–D, F–H, J–K, M–T, W–Z) {10}. The first may be situated on the dorsal (Fig. 6A–C, F–H, M, N, T) or ventral side of the axillary (Fig. 6D, J, K, O–S, W–Z) {11}. Several angles can be distinguished at this axillary. The α angle (between the legs of the body) is approximately 50° {15}. Another angle is created by the head and neck and measures approximately 90° (Fig. 6A, E, J–L, T–W) {16}. The internal angle between the neck and the distal leg is approximately 50° {17} and the external one 100°, respectively {18}.

#### Second axillary sclerite 2Ax (Fig. 3B—numbers in curly brackets; Fig. 7)

**Fig. 7:**
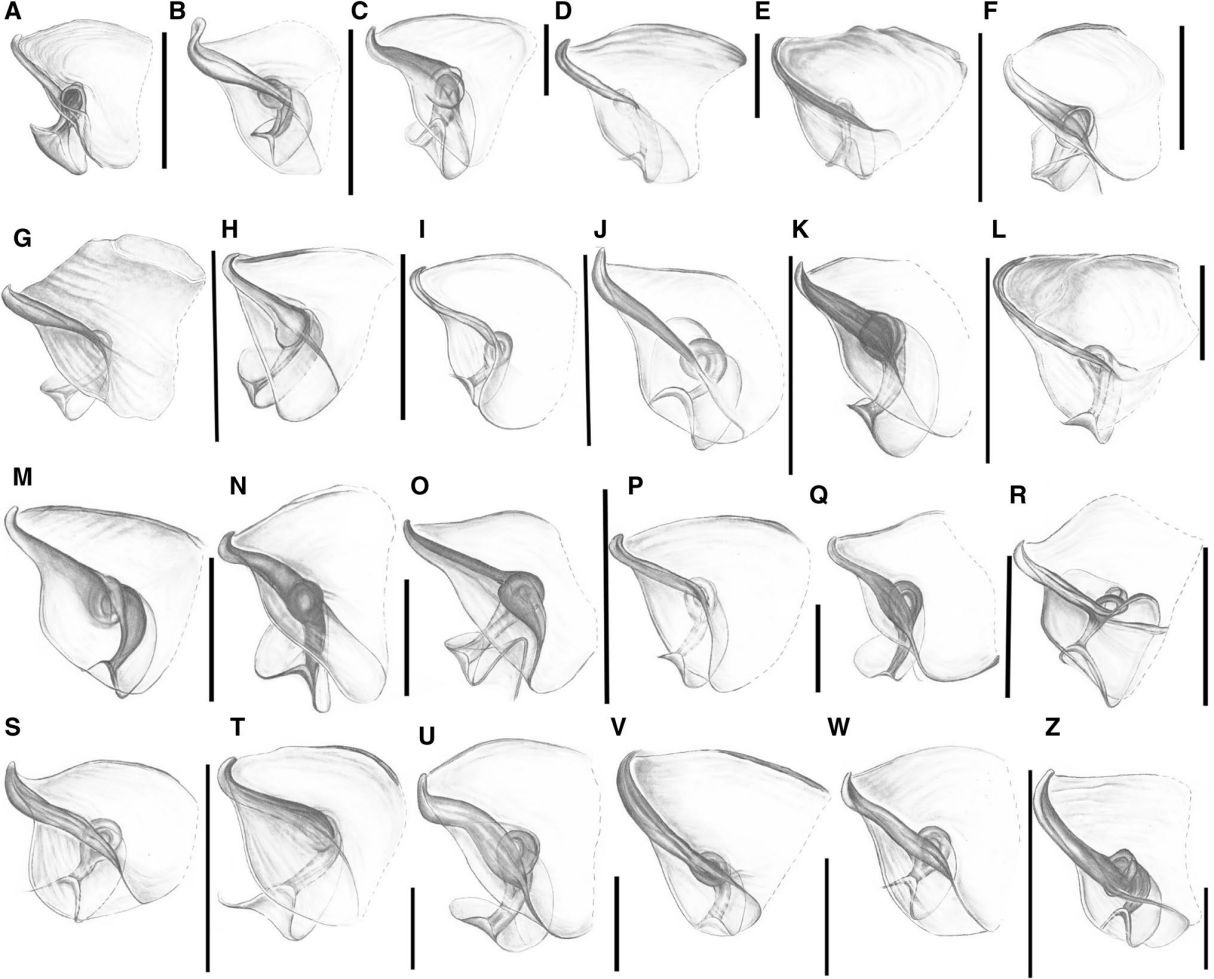
Schematic drawings of 2Ax. **A** *Adelges* sp.; **B** *Anoecia* sp.; **C** *Aphis* sp.; **D** *Chaitophorus* sp.; **E** *Cinara* sp.; **F** *Drepanosiphum* sp.; **G** *Eriosoma* sp.; **H** *Eucallipterus* sp.; **I** *Glyphina* sp.; **J** *Greenidea* sp.; **K** *Hormaphis* sp.; **L** *Lachnus* sp.; **M** *Macrosiphum* sp.; **N** *Mindarus* sp.; **O** *Neuqenaphis* sp.; **P** *Pemphigus* sp.; **Q** *Phloeomyzus* sp.; **R** *Phylloxera* sp.; **S** *Phyllaphis* sp.; **T** *Prociphilus* sp.; **U** *Thelaxes* sp.; **V** *Trama* sp.; **W** *Tuberculatus* sp.; **Z** *Tuberolachnus* sp.; scale bar 0.025 mm, except for (*R*) = 0.0125 mm

The outline of this sclerite may be triangular (Fig. 7A–F, H, L–M, U–W), oval (Fig. 7I, P, S, T) or trapezoid (Fig. 7G, J, K, N–O, Q, R, W–Z) {19}. One unique feature of this axillary is a ventral outgrowth, which is always curved on the dorsal side (Fig. 7A–Z) {20}. The degree of sclerotization may vary {21}. The connection between the ventral outgrowth and the middle of axillary may also be more (Fig. 7A–C, F, H–K, M–O, Q, R, U, V, Z) or less sclerotized (Fig. 7D–E, G, L, P, S, T, W) {22}. The site of this rounded element may also be different—in the middle of the axillary (Fig. 7A, K, O) or closer to the proximal (Fig. 7D, G, N, P, Q, S–U, W) or distal (Fig. 7B, C, E, F, H–J, L, M, R, V, Z) edge {26}. The proximal edge of the axillary, which faces the 1Ax, is mostly thick (Fig. 7B, C, F–H, K, M, N, Q–Z), rarely thin (Fig. 7A, D, E, I, J, L, O, P) {23} and is curved up on the outside (Fig. 7B–D, G, J, K, M, O, P, S) or smoothly passes through the rest of the membrane (Fig. 7A, E, F, H, I, L, N, Q, R, T–Z) {24}. The relationship between the length of the proximal edge {25″} and the length of the ventral outgrowth can be measured {25′}—the length may be the same (Fig. 7B, K, N) or the ventral outgrowth may be shorter than the proximal edge (Fig. 7A, C–J, L, M, O–Z) {25}. The membranous part of the axillary, which faces the wing membrane may create a flat, smooth surface (Fig. 7B, F–K, N, O, Q, R, U, W–Z) or may look like a bowl-like cavity (Fig. 7A, C–E, L, M, P, S, T, V) {27}. Additionally, the ventral outgrowth may be surrounded by the membrane (Fig. 7B, V–W); alternatively, it may even surround the membrane itself (Fig. 7D, E, I, J, L, M, P, R–T, Z) {28}.

#### Third axillary sclerite 3Ax (Fig. 3C—numbers in curly brackets; Fig. 8)

**Fig. 8:**
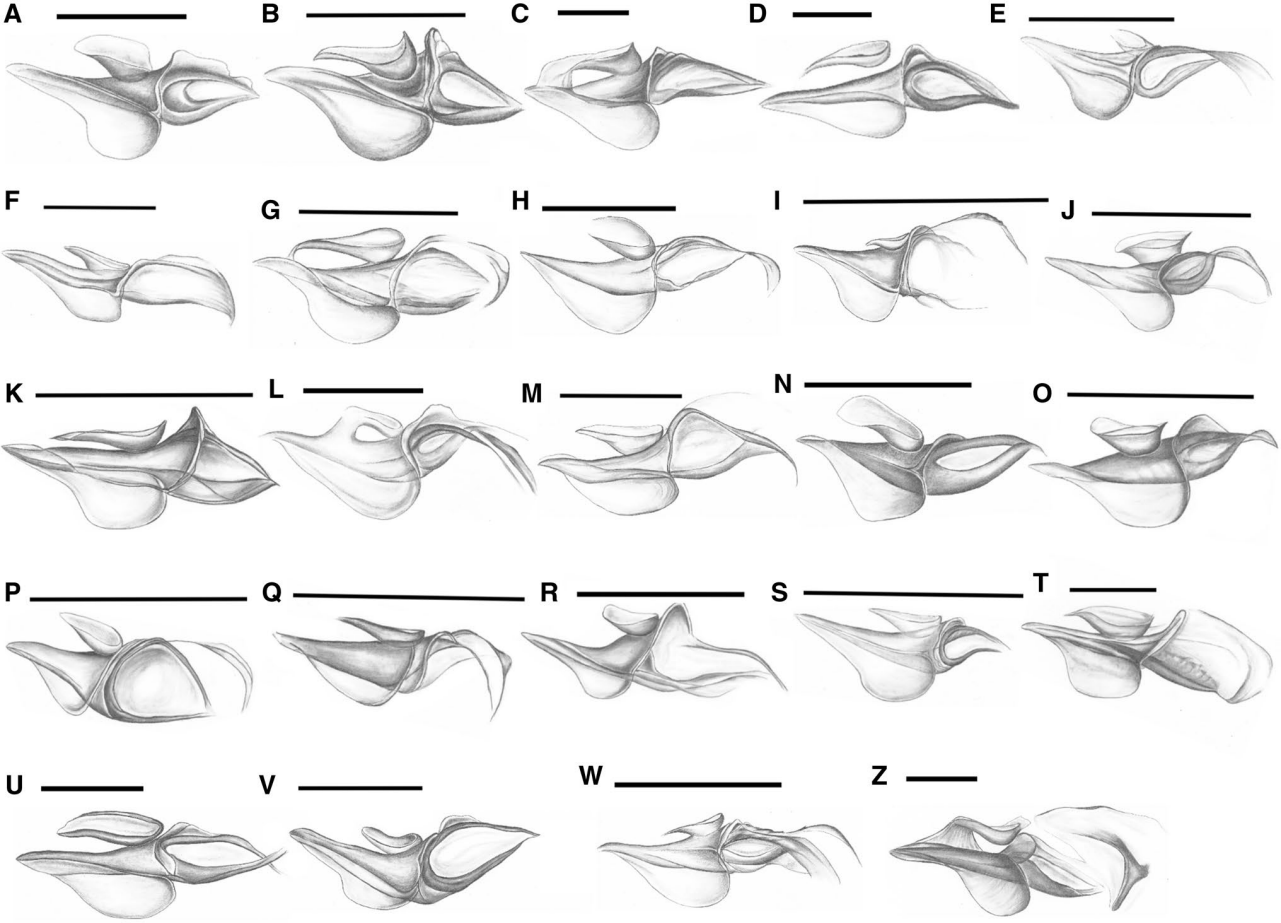
Schematic drawings of 3Ax. **A** *Adelges* sp.; **B** *Anoecia* sp.; **C** *Aphis* sp.; **D** *Chaitophorus* sp.; **E** *Cinara* sp.; **F** *Drepanosiphum* sp.; **G** *Eriosoma* sp.; **H** *Eucallipterus* sp.; **I** *Glyphina* sp.; **J** *Greenidea* sp.; **K** *Hormaphis* sp.; **L** *Lachnus* sp.; **M** *Macrosiphum* sp.; **N** *Mindarus* sp.; **O** *Neuqenaphis* sp.; **P** *Pemphigus* sp.; **Q** *Phloeomyzus* sp.; **R** *Phylloxera* sp.; **S** *Phyllaphis* sp.; **T** *Prociphilus* sp.; **U** *Thelaxes* sp.; **V** *Trama* sp.; **W** *Tuberculatus* sp.; **Z** *Tuberolachnus* sp.; scale bar 0.025 mm, except for (*R*) = 0.0125 mm

The entire plate may have the form of a triangle (Fig. 8E, S, V) or trapezoid (Fig. 8A–D, F–R, T, U, W–Z) {29} and consists of three elements—a proximal part close to the wing base (Fig. 3C: A), a distal part that faces the wing membrane (Fig. 3C: B) and an anterior bowl-like element (Fig. 3C: C). Proximal part usually has posterior membrane (Fig. 8A–Z) {30}. The proximal and distal parts are separated by a thin membrane (X) that is always on the ventral side of the axillary, although this transition may be very smooth (Fig. 8E, F, H–J, N, P, Q, S, U–W) at the level of the axillary or in the form of a high arc (Fig. 8A–D, G, K–M, O, R, T) {39}. The boundary between the proximal and distal parts is determined by this thin membrane. Therefore, it is possible to compare lengths of those elements {40}. The anterior bowl-like element, if present {34}, differs in its length, position and connection to the rest of the axillary. It may be long (Fig. 8B–D, G, K, M, N, Q, U) or short (Fig. 8A, E, F, H–J, L, O, P, R–T, V–W) {35} and may be situated near the thin membrane (X) (Fig. 8E, F, H–J, L, M, P–S, U, V, Z) or in the middle of the upper surface (Fig. 8A–D, G, K, N, O, T, W) {36}. The connection between this part and the rest of the axillary in most of the species is executed by the sitting position of this element {37}. Moreover, it may be connected by a thin (Fig. 8G) or wide membrane (Fig. 8C, L, Z) at the proximal edge {38}. The proximal part of 3Ax has a differently shaped membrane on its posterior margin. It is always rounded and may have a small indentation near the wing base {31}. The distal element of the third axillary may end differently. For some species, the ending of a sclerite consists of thick (Fig. 8A–E, J–L, N, O, S, U–Z) or thin arms (Fig. 8F–I, M, P–R) {32}. A wide (Fig. 8G, I, K, M, P, V, Z) or narrow (Fig. 8A–F, H, J, L, N, O, R, S, U, W) aperture is usually visible in the distal part {33}.

### Axillaries of *Orthezia urticae* (Fig. 3—numbers in curly brackets; Fig. 9)

The entire first axillary may have the shape of trapezium {1} and the head and the body can be easily distinguished. The neck and the body form a single element, and therefore many of its features cannot be described. The head is short, parallel to the body {3} and curved upward {6}. The ending of the head is sharp {5}. There is no visible connection between the head and the neck. The latter has the same width along its entire length {8}. The body of the axillary consists of a completely sclerotized membrane that is in the form of two thin legs that are equal in length {12} and are opened at a right angle. There is no outgrowth on the distal leg. The angle between the head and the neck is larger than 90° {16}.

The second axillary is triangular in shape {19} and does not have a ventral outgrowth {20}. The proximal edge of this axillary is thin {23}. The posterior edge faces the third axillary and they are only connected by a thin membrane.

The third axillary has the shape of a trapezoid {29} and is wider than higher. The posterior wall is a plane that has no posterior membrane {30}. On the distal end, which is a simple membrane, there is no aperture. The anterior part of the axillary also has no anterior outgrowth {34}. The middle of the axillary edge of the membrane (X) extends upwards toward the ventral side {39}. The proximal and distal parts are determined by the boundary membrane and the distal one is longer than the proximal one. Moreover, the distal part of 3Ax is situated parallel to the rest of the axillary. All of the axillaries are strongly sclerotized.

**Fig. 9:**
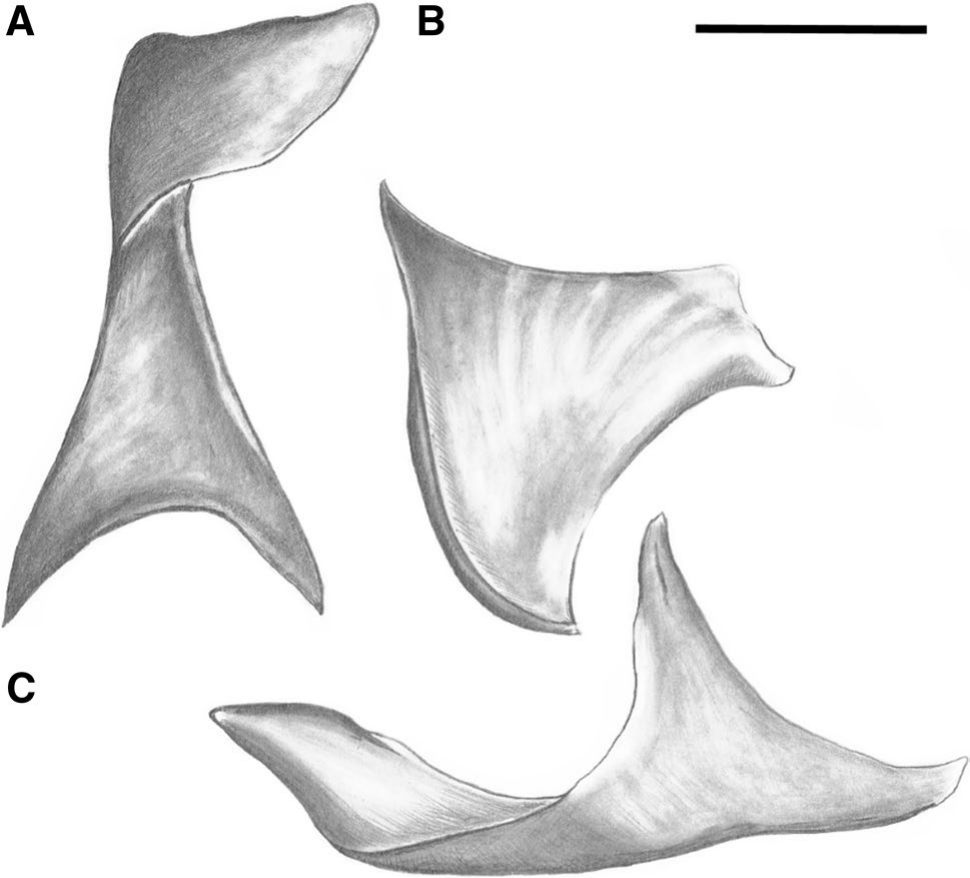
Schematic drawings of the axillary sclerites of *Orthezia urticae*. Scale bar 0.025 mm

### Comparative morphology between axillary sclerites among studied insects

The basic form of all of the axillaries among aphids is similar, but the degree of this similarity is lesser than would be expected in insects that belong to one infraorder. Taxonomic diversity, thorax construction as well as different environment of live (ex. in galls) may suggest differences between axillaries among aphids. The first axillary is divided into a head, neck and body parts in most of the studied species with one exception—*Drepanosiphum* sp., in which the head is shortened (char. 3:b) (Fig. 6F). In most cases the head is elongated and parallel to the body (3:a). Most aphid genera are characterized by a trapezoid shape of 1Ax (1:b). Only in *Aphis* sp. (Fig. 6C) it is most commonly in the shape of a parallelogram and is very strongly sclerotized (2:a). A membranous connection is very common between the head and neck (4:a), and only *O. urticae* does not have it (Fig. 9A). The ending of the head is mostly extended (5:c) and parallel to the body (6:b). The neck of 1Ax is shorter than the head (7:a) in most aphid species except for *Neuqenaphis* sp. (Fig. 6O), in which the neck and head are equal in length or for *Macrosiphum* sp. (Fig. 6M) and *Phloeomyzus* sp. (Fig. 6Q), in which the neck is shorter. Moreover, the width of the neck is the same along its entire length (8:a) and the neck is also slightly bent outward (9:a), which is common in the genera studied. There is no outgrowth of the body in *Cinara* sp. (Fig. 6E), *Glyphina* sp. (Fig. 6I), *Lachnus* sp. (Fig. 6L) and *Trama* sp. (Fig. 6V) (10:b). If present, it is closer to the neck (10:a) in more than half of genera studied and situated on the ventral side of the body (11:b). In half of the genera, the body is a uniform element (12:a). Alternatively, thin legs are observed (12:b). The length of the body legs (14) is correlated with the previous one, therefore genera that are described with character 12:a have the state b for character 14. Most specimens did not have a proximal outgrowth (13:a). The angles that were measured are in all genera rather big. The predominant angle between the legs of the body is larger than 50° (15:a), while between the head and neck it is 90° (16:a). The internal angle between the neck and distal leg is slightly higher than 50° (17:a), and in only three cases [*Phyllaphis* sp. (Fig. 6S), *Tuberculatus* sp. (Fig. 6W) and *Tuberolachnus* sp. (Fig. 6Z)] it was less than 50°. The external angle on the boundary neck-distal leg was more than or equal to 100° (18:b) for most of the genera examined.

The second sclerite mostly had a triangular shape (19:a), and a ventral outgrowth was always present (20:a) in aphids and absent in *O. urticae* (20:b). This sclerite was usually strongly sclerotized (21:a). More than half of the specimens had a strongly attached ventral outgrowth (22:a). This element is usually surrounded by the membrane (28:c) except for three species [*Anoecia* sp. (Fig. 7B), *Trama* sp. (Fig. 7V) and *Tuberculatus* sp. (Fig. 7W)] (28:b). The proximal edge of 2Ax was mostly thick (23:b) and ended smoothly by passing through the rest of the membrane (24:a). The ventral outgrowth of the axillary was somewhat shorter than the proximal edge (25:a), although the length was the same (25:a) for *Anoecia* sp. (Fig. 7B), *Hormaphis* sp. (Fig. 7K) and *Mindarus* sp. (Fig. 7N). A rounded element was usually present closer to the distal edge (26:c), except in *Adelges* sp. (Fig. 7A), *Hormaphis* sp. (Fig. 7K) and *Neuqenaphis* sp. (Fig. 7O) (26:a), in which this element was situated in the middle. A flat surface was usually created by the membranous part of axillary (27:b).

The third axillary in most studied species was trapezoid in shape (29:a). Only in *Cinara* sp. (Fig. 8E), *Phyllaphis* sp. (Fig. 8S) and *Trama* sp. (Fig. 8V) the shape was triangular. Axillary had always posterior membrane (30:a), which was mostly indented (31:b). The distal part of 3Ax ended with arms, which, in most cases, were thick (32:a), except for *Prociphilus* sp. (Fig. 8T) in which the distal part was a plane membrane (Fig. 8T). A narrow aperture was usually present in the membrane of the distal part (33:a), but in two cases, *Phloeomyzus* sp. (Fig. 8Q) and *Prociphilus* sp. (Fig. 8T), there was no membrane at all. An anterior outgrowth was present in all of the aphids (34:a) while the representative species for coccids did not have this element (34:b). Anterior outgrowth was rather short (35:a) and was situated very close to the edge of the membrane near the distal aperture (36:b). In only one case, *Chaitophorus* sp. (Fig. 8D), there was a lack of a connection between this outgrowth and the anterior part of 3Ax (37:b). Moreover, proximal edge of the anterior outgrowth and the proximal part of 3Ax, usually had no connection (38:a), although there was a thin membrane between them in *Eriosoma* sp. (Fig. 8G) (38:b), and the membrane was wide (38:c) in *Aphis* sp. (Fig. 8C), *Lachnus* sp. (Fig. 8L) and *Tuberolachnus* sp. (Fig. 8Z). The X membrane in the middle of the third axillary was usually flat and extended to the ventral side (39:b). It also marked boundary between the proximal and distal parts of this sclerite and in most cases had a proximal part that was longer than the distal one (40:a). In the case of *Mindarus* sp. (Fig. 8N) and *Phylloxera* sp. (Fig. 8R), the proximal part was shorter than the distal one (40:c). The additional character 41 had state b in coccid representative, *O. urticae* and in such aphid species as: *Glyphina* sp. (Fig. 8I), *Phloeomyzus* sp. (Fig. 8Q), *Phylloxera* sp. (Fig. 8R) and *Thelaxes* sp. (Fig. 8U), because only in those four cases wings fold flat over the abdomen in rest.

## Discussion

**Fig. 10:**
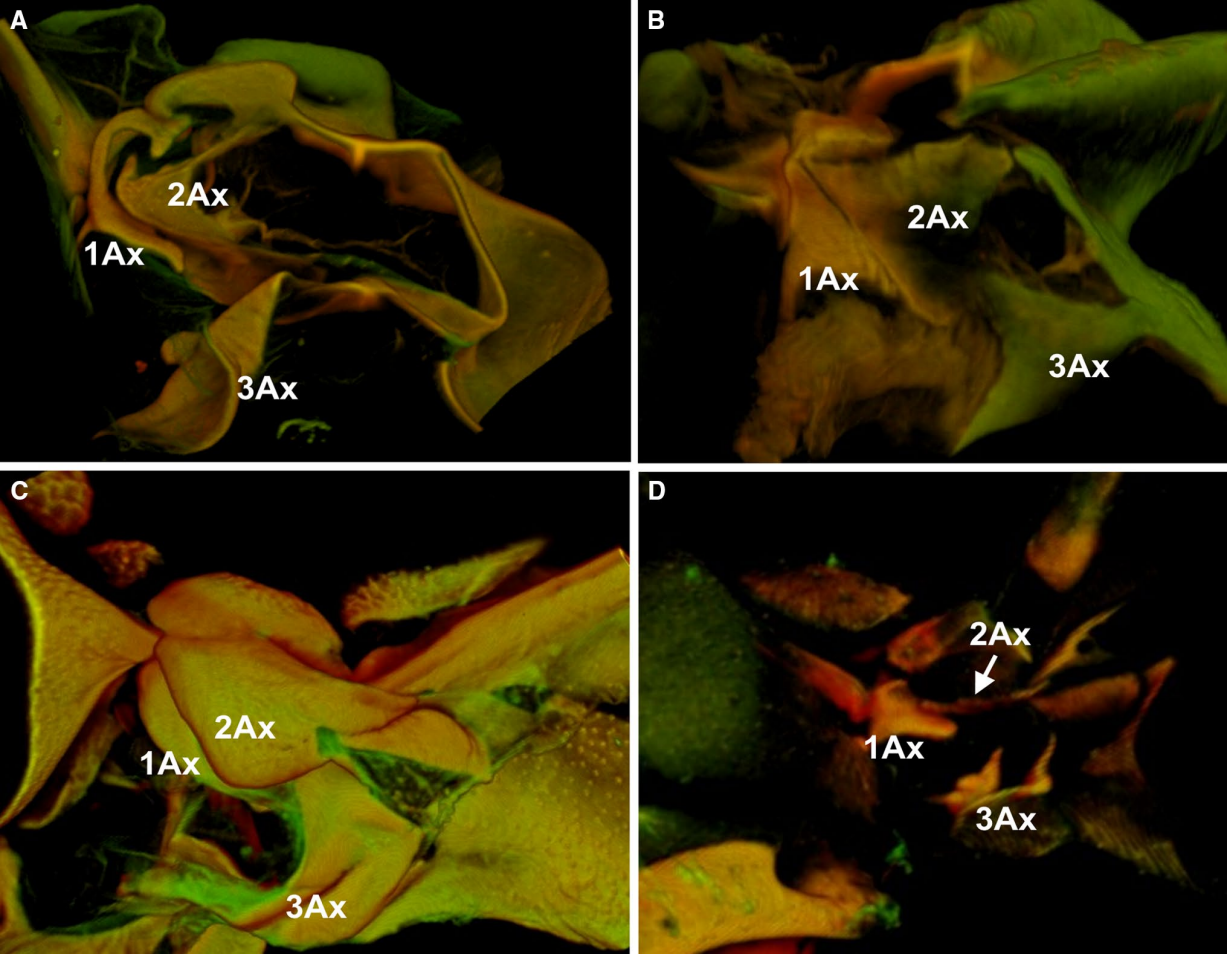
Multiphoton scanning microscopy showing the forewing articulation: **A** *Aphis fabae* (Scopoli); **B** *Orthezia urticae* (Linnaeus); **C** *Cacopsylla mali* (Schmidberger); **D** *Aleyrodes proletella* (Linnaeus)

Comparing these results with previously obtained (Franielczyk and Wegierek 2016), one finds a pronounced variability of the axillary sclerites architecture within one suborder, Sternorrhyncha. Between all four infraorders analyzed, there are four independent trends in axillary sclerites architecture. Nonetheless, detailed analysis enables to distinguish morphological features of sclerites which show similarity between aphids + coccids (first group) and psyllids + aleyrodids (second group) (Fig. 10). First group can be characterized with such features as: anterior tip of 1Ax curved around anterior end of 2Ax; 2Ax not overlapping 1Ax; flat humeral plate and presence of the connection between 1Ax/2Ax. The characteristic features of the second group are: large, globular tegula; tubercle-like humeral plate; presence of the connection between 1Ax/2Ax and lack of anterior tip of 1Ax around anterior tip of 2Ax.

However, particularly in Aphidomorpha infraorder, apart from small discrepancies in the axillary structures, the general model of axillaries is similar. Thus, the differentiating factors cannot be described. The complexity of spatial organization of the structures is presented in the animation of *Aphis fabae* (Aphididae) (Online Resource 2).

Our data indicate that the way the wing folds is not determined by the morphology of axillary sclerites but rather by the architecture of the thorax. No clear relationship between morphology of the axillary sclerites and the wing folding could be established. It is surprising that the wing base architecture differs among aphid species and the architecture of sclerites enforces their description using numerous morphological features. Exceptionally, no significant differences in wing base could be observed between viviparous (Aphididae) and oviparous (*Adelges* sp., *Phylloxera* sp.) species, which are considered to belong to older genera (von Dohlen and Moran 2000).

As indicated by Zhao et al. (2014), morphometric analysis of the wing base may be important for a phylogenetic study. However, the spatial architecture of aphids wing base described by us does not seem to be sufficient tool for conducting phylogenetic analysis among aphids. We show that it is difficult to indicate morphological similarities in axillary sclerites construction, although the affinities within these taxa have already been established (based on morphological and genetic studies). It might be speculated, that the shape of axillary sclerites in aphids evolved as the result of the environmental pressure, however, determination of the most important factors impacting this evolution needs further studies.

Here we present for the first time detailed description of the relationship between the morphology of the forewing axillary sclerites and the way the wings fold among 24 aphid genera as compared to a representative of coccids. Further detailed analysis concerning other groups of Hemiptera, needs to be conducted. So far, this part of insect body is unlikely to be investigated in fossil insects. New methods are needed to study preserved wing base in fossil material (both as impressions on the rock and inclusions in amber). For now, even microCT is not yet sufficient to study such small insects as Sternorrhyncha representatives (Franielczyk-Pietyra, Wegierek unpublished). That is the reason why paleontological criterium is not use in this insect group for phylogenetic research on this subject.

## Electronic supplementary material

Below is the link to the electronic supplementary material.

Online Resource 1. Taxa examined (PDF 84 KB)

Online Resource 2. Animation presenting the 3D structure of *Aphis fabae* sclerites (MP4 7507 KB)
