# Supplementary material for: Is there a relationship between the morphology of the forewing axillary sclerites and the way the wing folds in aphids (Aphidomorpha, Sternorrhyncha, Hemiptera)?
Source: Zoomorphology. 2017 Nov 30;137(1):105–17. doi: 10.1007/s00435-017-0390-7 (PMC5847081; doi:10.1007/s00435-017-0390-7)
Supplement: Supplementary file 1 — Online Resource 1. Taxa examined (PDF 84 KB) [file 435_2017_390_MOESM1_ESM.pdf]

Table 1. Examined taxa (after Heie and Wegierek 2009, modified)

**Superfamily**	**Family**	**Subfamily**	**Tribe**	**Species**	**Locality**
Adelgoidea	Adelgidae			*Adelges sp.* (Vallot)	Ustroń PL N49°43`W18°48`
Phylloxeroidea	Phylloxeridae			**Phylloxera sp.* (Boyer de Fonscolombe)	Katowice PL N50°15`E19°01`
Aphidoidea	Eriosomatidae	Eriosomatinae		*Eriosoma sp.* (Leach)	Katowice PL N50°15`E19°01`
		Pemphiginae		*Pemphigus sp.* (Hartig)	Katowice PL N50°15`E19°01`
		Prociphillinae		*Prociphilus sp.* (Koch)	Katowice PL N50°15`E19°01`
	Hormaphididae	Hormaphidinae		*Hormaphis sp.* (Osten-Sachen)	Seoul South Korea N37°33`E126°59`
	Phloeomyzidae			**Phloeomyzus sp.* (Horvath)	Piekary Śląskie PL N50°22`E18°56`
	Thelaxidae			**Thelaxes sp.* (Westwood)	Piekary Śląskie PL N50°22`E18°56`
				**Glyphina sp.* (Koch)	Katowice PL N50°15`E19°01`
	Anoeciidae			*Anoecia sp.* (Koch)	Piekary Śląskie PL N50°22`E18°56`
	Drepanosiphidae	Mindarinae		*Mindarus sp.* (Koch)	Piekary Śląskie PL N50°22`E18°56`
		Drepanosiphinae		*Drepanosiphum sp.* (Koch)	Katowice PL N50°15`E19°01`
		Spicaphidinae		*Neuqenaphis sp.* (Blanchard)	Chile South America S39°10`W71°42`
		Phyllaphidinae		*Phyllaphis sp.* (Koch)	Katowice PL N50°15`E19°01`
		Calaphidinae		*Tuberculatus sp.* (Mordvilko)	Katowice PL N50°15`E19°01`
				Eucallipterus sp. (Schouteden)	Ustroń PL N49°43`W18°48`
		Chaitophorinae	Chaitophorini	*Chaitophorus sp.* (Koch)	Katowice PL N50°15`E19°01`
	Greenideidae	Greenideinae	Greenideini	*Greenidea sp.* (Schouteden)	Seoul South Korea N37°33`E126°59`
	Aphididae	Aphidinae	Aphidini	*Aphis sp.* (Linnaeus)	Piekary Śląskie PL N50°22`E18°56`
		Macrosiphinae		*Macrosiphum sp.* (Oestlund)	Piekary Śląskie PL N50°22`E18°56`
	Lachnidae	Lachninae	Lachnini	*Lachnus sp.* (Burmeister)	Katowice PL N50°15`E19°01`
				Tuberolachnus sp. (Mordvilko)	Katowice PL N50°15`E19°01`
			Tramini	*Trama sp.* (von Heyden)	Katowice PL N50°15`E19°01`
		Eulachninae		*Cinara sp.* (Curtis)	Borne Sulinowo PL N53°34`E16°32`
Coccoidea	Ortheziidae			**Orthezia urticae* (Linnaeus)	Goczałkowice Zdrój PL N49°56`E18°58`

* wings fold flat in the rest
